# First-in-human trial using mixed-reality visualization for patient setup during breast or chest wall radiotherapy

**DOI:** 10.1186/s13014-024-02552-0

**Published:** 2024-11-18

**Authors:** Perry B. Johnson, Julie Bradley, Samsun Lampotang, Amanda Jackson, David Lizdas, William Johnson, Eric Brooks, Raymond B. Mailhot Vega, Nancy Mendenhall

**Affiliations:** 1 University of Florida Health Proton Therapy Institute, Jacksonville, FL USA; 2 University of Florida College of Medicine, Gainesville, FL USA

**Keywords:** Mixed-reality, HoloLens 2, Surface-guided radiotherapy, Breast radiotherapy

## Abstract

**Background:**

The purpose of this study is to assess the feasibility of mixed-reality (MixR) visualization for patient setup in breast and chest wall radiotherapy (RT) by performing a first-in-human clinical trial comparing MixR with a 3-point alignment.

**Methods:**

IRB approval was granted for a study incorporating MixR during the setup process for patients undergoing proton (*n* = 10) or photon (*n* = 8) RT to the breast or chest wall. For each patient, MixR was utilized for five fractions and compared against another five fractions using 3-point alignment. During fractions with MixR, the patient was aligned by at least one therapist wearing a HoloLens 2 device who was able to guide the process by simultaneously and directly viewing the patient and a hologram of the patient’s surface derived from their simulation CT scan. Alignment accuracy was quantified with cone-beam CT (CBCT) for photon treatments and CBCT plus kV/kV imaging for proton treatments. Registration time was tracked throughout the setup process as well as the amount of image guidance (IGRT) utilized for final alignment.

**Results:**

In the proton cohort, the mean 3D shift was 0.96 cm using 3-point alignment and 1.18 cm using MixR. An equivalence test indicated that the difference in registration accuracy between the two techniques was less than 0.5 cm. In the photon cohort, the mean 3D shift was 1.18 cm using 3-point alignment and 1.00 cm using MixR. An equivalence test indicated that the difference in registration accuracy was less than 0.3 cm. Minor differences were seen in registration time and the amount of IGRT utilization.

**Conclusions:**

MixR for patient setup for breast cancer RT is possible at the level of accuracy and efficiency provided by a 3-point alignment. Further developments in marker tracking, feedback, and a better understanding of the perceptual challenges of MixR are needed to achieve a similar level of accuracy as provided by modern surface-guided radiotherapy (SGRT) systems.

**Trial registration:**

ClinicalTrials.gov, UFHPTI 2015-BR05: Improving Breast Radiotherapy Setup and Delivery Using Mixed-Reality Visualization, NCT05178927.

**Supplementary Information:**

The online version contains supplementary material available at 10.1186/s13014-024-02552-0.

## Background

Mixed-reality (MixR) visualization describes the blending of physical and virtual environments using immersive technology. Advanced techniques utilize head-mounted displays that are capable of tracking the environment and displaying digital projections (i.e., holograms) at specific locations based on the current perspective of the user. These projections are persistent and exist in relation to either known objects (i.e., marker-based tracking) or environmental features, a process known as visual simultaneous localization and mapping (VSLAM – i.e., marker-less tracking). MixR is an emerging domain in medicine with current applications in education and training, telemedicine, procedural planning, and surgical navigation [1–4].

In our prior work, MixR was adapted as a tool for posture correction and alignment during the setup of a patient receiving radiotherapy (RT) [5]. In this technique, a user wearing a head-mounted display like the HoloLens 2 (Microsoft Corp., Redmond, WA) is able to simultaneously and directly view a patient and a hologram of the patient’s surface derived from their simulation CT scan. The hologram provides a visual reference for the exact posture needed during treatment and is initialized in relation to the isocenter of the treatment machine. Thus, by matching a patient to their hologram during setup, the correct posture is achieved while subsequently registering the patient with the treatment device. A MixR application (HoloMatch v1) was developed to implement this technique using the HoloLens 2. In preliminary testing with phantoms, the system demonstrated registration error of 0.3 cm for rigid alignment and displayed unique advantages for dealing with the type of non-rigid alignment often encountered during patient setup.

The purpose of the present study was to extend this testing to humans by performing a first-in-human clinical trial (pilot study) to assess the feasibility of the technique for patient alignment. In the trial, registration error, registration time, and amount of image guidance (IGRT) used during the registration process were compared between MixR and a traditional 3-point alignment using surface marks and room lasers. The study involved patients undergoing either photon or proton RT for treatment of the whole breast or chest wall, a challenging anatomical site based on the independent movement of the local anatomy and the deformability of a pendulous (for breast) target. The results are discussed in comparison to both surface guided radiotherapy (SGRT) and recent studies involving MixR for patient setup. As this was a pilot study, a secondary purpose was to assess the trial design to better understand its limitations and define areas for improvement, as well as discovering new aspects about MixR-based alignment that may be worth investigating in a larger trial in the future. Such a trial would benefit from lessons learned in the present study.

## Methods

### Software development

HoloMatch v2 was developed in Unity v2021.1.19f1 (Unity Technologies, San Francisco, CA) using Microsoft’s Mixed Reality Toolkit Foundation v2.7.2 and the Mixed Reality OpenXR Plugin - v1.4.0 (Microsoft Corp., Redmond, WA). In this version, a new Python script was written to automate the conversion of a DICOM (Digital Imaging and Communications in Medicine) RT plan file and structure file into a formatted OBJ file representative of a patient’s body surface and associated treatment isocenter. This file can be visualized through the HoloLens 2 as a holographic object and is readily loaded to the device through a windows-based file transfer process. A text file is also generated with the patient’s name and medical record number which can be linked to setup photos also loaded to the device. A new holographic menu system was developed for importing the reference surface, selecting the patient and treatment plan, displaying setup photos, and adjusting the hologram’s appearance via selection of different shaders (scripts that govern holographic rendering). The menu system can be accessed using hand gestures or voice commands and was designed as such that any opened windows tag-along with the user and remain within their field of vision without being locked to the position of the user’s head, as encountered in traditional heads-up displays. Setup photos can be placed anywhere within the immediate space and sized according to the preference of the user. For this work, a Fresnel-derivative shader was used to define the hologram’s appearance. This semi-transparent shader highlights edges and surfaces with high angles of incidence and was one of the most preferred shaders as indicated by users during an initial study evaluating different holographic visualization techniques for MixR-based alignment [6].

**Fig. 1:**
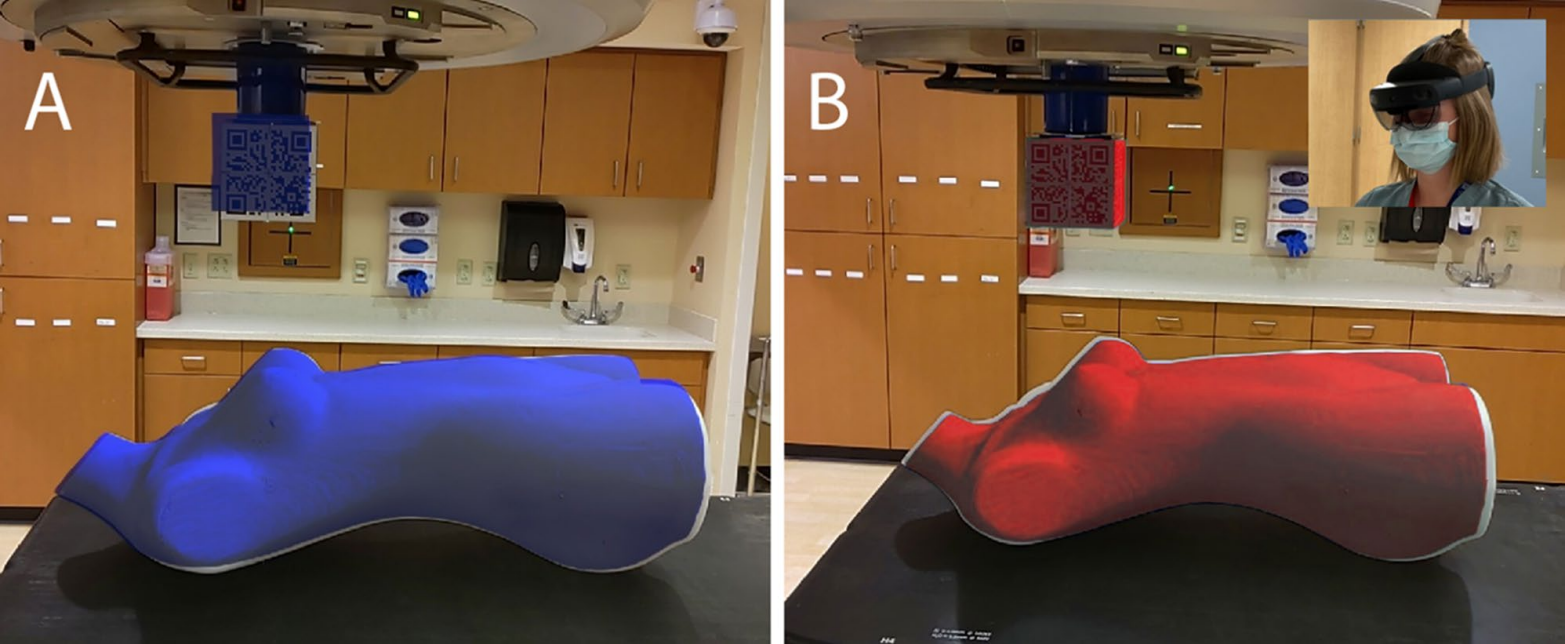
**Panel A**; The blue holographic overlays of both the patient and QR cube indicate the use of marker-less (VSLAM) tracking. Although the phantom has been aligned to its hologram, the phantom’s position is incorrect as there is a clear difference between the QR cube and it’s holographic twin. This can happen if there are changes within the local environment that affect features being tracked by the VSLAM algorithm. **Panel B**; To correct this, the user must switch to marker-based tracking as indicated by the red holographic overlays. Here, The misalignment of the QR cube has been fixed, and the true misalignment of the patient can be seen

The primary function of HoloMatch is to guide patient alignment during the RT setup process. This is done by first initializing the patient hologram at the machine isocenter based on the recognition of a QR-coded cube, e.g., the marker needed for marker-based tracking. This physical marker is designed to attach to either a linear accelerator or proton gantry with a known offset between the center of the cube and the isocenter of the machine. The HoloLens 2 recognizes the cube via marker-based tracking, and the patient hologram is then displayed at the correct location based on the known relationships between the physical cube, the machine isocenter, and the patient isocenter. A hologram of the cube is also added to the scene to provide visual feedback for this registration process, i.e., if the hologram of the cube is not aligned with the physical cube then something is wrong in the tracking process. During RT setup, a patient is matched to their hologram as visualized by a user wearing the HoloLens 2 and running the HoloMatch application. Once the hologram is initialized, the HoloLens 2 uses marker-less tracking to constantly track environmental features and update the holographic projection based on the perspective of the user (see VSLAM), thus enabling freedom of movement to view the patient and hologram from multiple angles without the need to constantly focus on the QR cube, as is needed with marker-based tracking. If the local environment changes enough, the holographic scene may drift because the environmental features used by the HoloLens 2 for tracking are no longer static (Fig. 1A). To account for this, the user can temporarily switch to marker-based tracking using a voice command to correct any perceived drift in hologram-to-machine registration as indicated by the physical/holographic cube overlay (Fig. 1B). For quick reference as to which tracking mode is enabled, the patient hologram appears red when using marker-based tracking and blue when using marker-less tracking.

### Clinical trial design

This study focused on patients receiving either breast or chest wall radiotherapy. These anatomical sites were chosen based on the challenges posed by the independent movement of the local anatomy (e.g., torso, breast, arms, shoulders, head and neck), which highlight the utility of a surface-based alignment versus a traditional 3-point alignment. Breast and chest wall radiotherapy is also a common treatment, making accrual easier. The study was also funded by the Florida Breast Cancer Foundation.

As this was a pilot study, patient selection was broad with the study being offered to any adult patient receiving breast or chest wall radiotherapy. Additionally, based on the way in which the study was designed, each patient served as their own control, thus eliminating the need to carefully apportion patients within arms associated with setup technique. Patients were, however, enrolled into different study groups based on whether they were receiving proton or photon therapy. This was done under the assumption that the results might be different within each group based on (1) a higher prevalence of intact breast patients are treated with photon therapy at our clinic, whereas proton therapy is more often used for chest wall cases, and (2) the setup tolerances are tighter with proton therapy, and at our clinic are known to involve more iteration during the setup process and more use of IGRT. Ultimately, the study included 18 female patients receiving either photon (8) or proton (10) RT for treatment of the whole breast or chest wall (Table 1). Ten patients also received treatment to regional lymph nodes (with the majority of those found in the proton group), and four patients had either an implant or expander in place at the time of the study. The median age for those included in the study was 53 years [age range, 31–77 years]. Each patient was consented and enrolled according to an IRB approved protocol. Since this was a pilot study, no formal power analysis was applied, and the choice of sample size was based on practicality.

**Table 1:** Demographic and clinical information for patients included in the trial. [Proteus Plus Gantry Treatment Room 2; P + GTR2]

Patient	Age	Weight (lb)	Anatomical Site	Reconstruction	Regional nodes	Machine
Proton cohort						
1	31	150	Left chest Wall	No	No	P + GTR2
2	70	133	Left chest Wall	No	Yes	P + GTR2
3	55	115	Left breast	No	Yes	P + GTR2
4	77	150	Left chest wall	No	Yes	P + GTR2
5	37	205	Left chest wall	Expander	Yes	P + GTR2
6	53	204	Left breast	No	Yes	P + GTR2
7	47	152	Right chest wall	Expander	No	P + GTR2
8	55	154	Right breast	No	Yes	P + GTR2
9	55	177	Left chest wall	Implant	Yes	P + GTR2
10	53	145	Left chest wall	No	Yes	P + GTR2
Photon cohort						
1	72	196	Left breast	No	No	TrueBeam
2	68	144	Left breast	No	No	TrueBeam
3	46	187	Right breast	No	No	TrueBeam
4	38	144	Left breast	No	No	TrueBeam
5	77	180	Right breast	No	No	TrueBeam
6	58	142	Left breast	No	Yes	Synergy
7	63	203	Left breast	No	No	TrueBeam
8	39	280	Right chest wall	Expander	Yes	Synergy

To control for setup variation which typically decreases after the first few treatment fractions, patients were stratified into two groups using an alternating method. In the first group, patients were positioned using 3-point alignment using surface marks during fractions 1–5, then using MixR for fractions 6–10. During setups using MixR, surface marks were disregarded. For the second group, the order was reversed, using MixR for fractions 1–5 and 3-point alignment for fractions 6–10. In this way, each patient served as their own control. In all cases, the initial patient alignment was verified using cone beam computed tomography (CBCT). The magnitude of the resulting shift was recorded, and if further adjustment was necessary, IGRT (including CBCT and/or orthogonal x-rays, i.e., kV-pairs) was exclusively utilized thereafter. The number of repeated CBCTs and/or kV-pairs, were recorded along with the shift magnitude. The number of repeat images is an indicator of how much iteration was used during the alignment process, and hence helps describe the amount of difficult encountered by the therapist during the setup procedure. The total number of CBCTs and kV-pairs collected during the alignment process is henceforth discusses as the “amount of IGRT”. A time log was created for each patient indicating the length of the initial setup and time taken to acquire subsequent imaging, i.e., registration time. The timer began once the patient was lying on the treatment couch and the therapists initiating the alignment process. The “initial registration time” was recorded once the initial setup was completed using either 3-point alignment or MixR but prior to CBCT validation. The “overall registration time” was recorded at the end of any subsequent imaging once the therapists were satisfied with the patient’s setup. During fractions with MixR, the HoloLens 2 was worn by at least one therapist, and in some cases also worn by a second individual, either a second therapist or a study investigator.

Patients receiving proton RT were treated using an IBA Proteus Plus Gantry (Ion Beam Applications, Louvain-la-Neuve, Belgium) capable of pencil beam scanning. Patients receiving photon RT were treated using either a Varian TrueBeam (Varian Medical Systems, Palo Alto, CA) or an Elekta Synergy (Elekta, Stockholm, Sweden). Custom mounts were designed using the accessory trays of each system to efficiently attach and remove the QR block during the setup process. Two steps were used to ensure the quality of the localization between the patient’s hologram and the machine isocenter. First, the room lasers were used to check that the physical cube was centered directly above the machine isocenter. Second, the hologram of the cube was checked to ensure that it correctly overlaid the physical cube. Through these two steps, the registration between the machine and the physical cube, and the registration between the physical cube and the holographic scene were verified.

Patients were positioned using an inclined wing board that was also visible as part of each patient’s hologram. Simulation, planning, and treatment were performed based on standard of care with only the initial setup changing based on the inclusion of MixR for certain fractions. Two patients in the photon cohort were treated using a breath hold technique implemented using an Active Breathing Control (ABC) device. The use of this device did not impede the MixR alignment process and the patient was aligned during consecutive breath holds by visualizing the patient’s holographic surface as derived from their breath hold CT scan. Each therapist who participated in the study went through a 30-minute training session using the HoloLens 2 device. During the session, users were instructed on how to use the device to align patients and practiced this workflow several times by aligning an anthropomorphic phantom with the isocenter of the TrueBeam and verifying it with CBCT.

For individual cases, data was averaged across a given set of five fractions to determine mean estimates of registration error and registration time for MixR and 3-point alignment and the paired difference of the two techniques. Van Herk’s specification for systematic and random error was further used to calculate the group systematic error (M) for a given parameter, as well as the standard deviation of the systematic (Σ) and random (σ) errors. Essentially, M is the mean of the means, Σ is the standard deviation of the means per patient, and σ is the root mean square of the standard deviation of all patients [7]. The results were assessed using a paired t-test (p-value < 0 0.05) and a two one-sided t-test (TOST) (p-value < 0.05), also known as an equivalence test. The null hypothesis for the paired t-test was that the mean paired differences across all measures of registration error and registration time was zero; the null hypothesis for the equivalence test was that these measures were significantly different than 0.3 cm and 5 min, respectively. These thresholds represent approximately 50% or less of the average 3D error or total registration time that might be expected and were thus chosen at the discretion of the study authors to represent feasibility for this pilot study. The amount of IGRT (CBCTs + kV-pairs) collected as part of the registration process was assessed subjectively since count data are not truly continuous.

## Results

Looking first at the effect of stratifying patients within a given modality based on the order in which MixR was applied, within the photon cohort the average 3D vector when using MixR was 1.10 ± 0.27 for fractions 1–5 and 0.97 ± 0.28 for fractions 6–10. Within the proton cohort the average 3D vector when using MixR was 1.36 ± 0.61 for fractions 1–5 and 1.24 ± 0.52 for fractions 6–10. Similarly, within the photon cohort the average 3D vector when using 3-point alignment was 1.31 ± 0.83 for fractions 1–5 and 1.06 ± 0.50 for fractions 6–10, and within the proton cohort was 1.19 ± 0.63 for fractions 1–5 and 1.00 ± 0.21 for fractions 6–10. According to these results, there was a consistent trend that matched the hypothesis that setup accuracy would improve for later fractions, though the patient numbers are limited and the uncertainty is high.

**Fig. 2:**
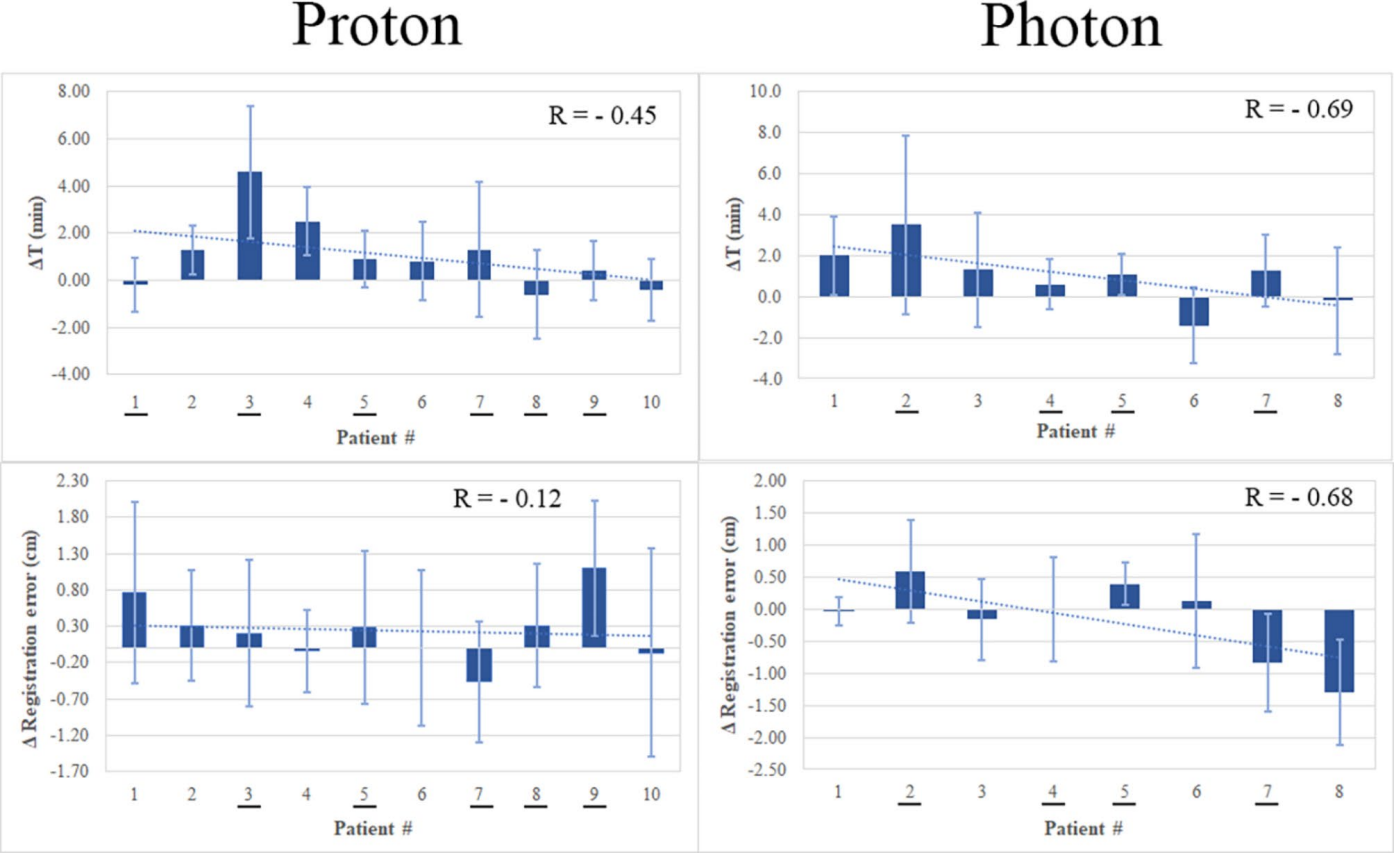
Top; Difference in the initial setup time when using mixed-reality (MixR) and 3-point alignment (ΔT = Initial_Setup_Time_MixR_ – Initial_Setup_Time_3 − point_) for each patient included in the study ordered by enrollment date within each cohort (earliest to latest). Bottom; Average difference in the 3D vector when using MixR and 3-point alignment (Δ = Registration_Error_MixR_ – Registration_Error_3 − point_) for each patient included in the study ordered by enrollment date within each cohort (earliest to latest). Error bars represent ± 1 standard deviation. Underlined numbers on the x-axis represent patients for which MixR was used during fractions 1–5

Figure 2 shows the difference in the initial setup time between the MixR and 3-point alignment for individual patients numbered by the order in which they enrolled in the study (ΔT = Initial_Setup_Time_MixR_ – Initial_Setup_Time_3 − point_). As seen, setup time when using the MixR technique did appear to decrease over the course of the study, with decreased setup time for patients enrolled into the study later compared to those patients enrolled at study initiation. The trend was similar between photon (*R* = ‒ 0.69) and proton (*R* = ‒ 0.45) cohorts, possibly indicating an increasing familiarity with the HoloLens 2 and MixR technique that occurred as the study progressed. The proton cohort took slightly longer to setup than the photon cohort, likely due to the presence of more complicated chest wall cases, longer CBCT acquisitions, and the smaller setup tolerance employed in proton RT. When averaged across all patients for a given modality, the initial setup time was one minute longer and the final setup time was two minutes longer when using MixR compared to 3-point alignment. However, this difference was not statistically significant according to a paired t-test. Furthermore, the results rejected the null hypothesis of the equivalence test indicating that the mean setup times were, statistically, within 5 min of each other. Table 2 provides the group means (average of five fractions then averaged across all patients) for directional shifts, azimuthal shifts, registration time, and the amount of IGRT usage. As seen in Table 2, the latter was identical in photon patients when comparing MixR and 3-point alignment and only slightly increased for MixR when making the same comparison for proton patients. Similar to overall setup time, and likely for the same reasons, more IGRT was reported in the proton cohort for both MixR and 3-point alignment setups.

**Table 2:** Group means, i.e., systematic error (M), standard deviation of the systematic error (Σ), and standard deviation of the random error (σ) for the directional and azimuthal shifts, as well as the magnitude of the 3D vector as categorized by modality and setup technique. Also highlighted are the group means and standard deviations for the number of CT or kV-pairs (image-guided radiotherapy; IGRT) acquired during the setup process for each patient, e.g., 1 CBCT + 2 kV-pairs = 3 IGRT (note that data is averaged across multiple fractions, hence the existence of decimal numbers). Also included are the group means and standard deviations for the initial and final registration times. The initial registration time represents the time point immediately following mixed-reality (MixR) or 3-point alignment, and the final registration time represents a time point at the conclusion of any further imaging needed to precisely align the patient for treatment

Modality	Approach		Initial Shift (cm/deg)														
			Lt/Rt				Ant/Post				Sup/Inf				3D Vector		
			M	Σ	σ		M	Σ	σ		M	Σ	σ		M	Σ	σ
Proton (*n* = 10)	3-point		-0.06	0.44	0.98		-0.07	0.28	0.53		-0.10	0.30	0.62		0.96	0.31	0.70
	MixR		-0.49	0.47	0.80		-0.18	0.55	0.89		-0.44	0.33	0.63		1.18	0.42	0.88
Photon (*n* = 8)	3-point		-0.37	0.83	0.43		-0.21	0.38	0.58		0.10	0.62	0.65		1.18	0.65	0.51
	MixR		-0.07	0.32	0.57		0.16	0.55	0.75		0.11	0.41	0.35		1.00	0.20	0.56
			**Rotation**				**Pitch**				**Roll**						
			M	Σ	σ		M	Σ	σ		M	Σ	σ				
Proton (*n* = 10)	3-point		0.26	0.22	0.83		0.06	0.37	0.75		0.01	0.57	0.88				
	MixR		-0.09	0.53	0.79		0.15	0.34	0.33		-0.17	0.42	0.66				
Photon (*n* = 8)	3-point		0.06	1.45	1.52		0.12	0.81	0.75		-0.15	0.97	0.93				
	MixR		-0.25	1.61	1.29		0.25	0.52	0.62		-0.36	1.21	1.16				
				**Registration Time (min)**							**IGRT (#)**						
			**Initial**				**Overall**										
			M	Σ	σ		M	Σ	σ		M	Σ	σ				
Proton (*n* = 10)	3-point		3.05	0.71	0.86		13.79	1.26	3.25		3.16	0.52	0.94				
	MixR		4.11	1.16	1.56		15.7	1.77	2.62		3.60	0.95	0.93				
Photon (*n* = 8)	3-point		3.34	1.04	0.99		8.80	2.38	3.28		1.10	0.21	0.50				
	MixR		4.36	1.71	2.19		10.54	2.52	3.05		1.11	0.16	0.40				

As noted the group means for the directional and azimuthal shifts, and the magnitude of the 3D vector are listed in Table 2. MixR appeared to do slightly better with pitch, roll, and rotation, and was very similar with regards to orthogonal shifts. No individual metric was significantly different according to a paired T-Test, except pitch, which showed slight advantage for the MixR technique in photon patients (p-value = 0.005). While the difference in registration error (Δ = Registration_Error_MixR_ – Registration_Error_3 − point_) generally decreased in photon patients as the study progressed (*R* = ‒ 0.68), this pattern was not present in the proton cohort (*R* = ‒ 0.12) (Fig. 2). Table 2 also highlights the variation in the data in terms of both systematic (Σ) and random (σ) errors. These data points were largely similar between the proton and photon cohorts. Table 3 also provides a list of recent studies that have assessed registration error when using 3-point alignment, and the variation in these studies is similar to that found in the present work.

**Table 3:** The reported accuracy of surface-guided radiotherapy (SGRT) for setups involving the breast, chest wall (CW) and/or regional lymph nodes. [Cone beam CT; CBCT]

Reference	Anatomical site	Patients	Verification	3D Error (range or σ) [cm]	
				3-Point	SGRT
Stanley et al. (2017)	Breast	> 200	CBCT	1.40 (0.70)	0.60 (0.20)
Kugele et al. (2019)	Breast	63	MV or kV/kV	0.42 (0-1.97)	0.24 (0-0.81)
	Breast/CW + Nodes	76		0.47 (0-1.87)	0.40 (0-1.35)
Hattel et al. (2019)	Breast/partial breast	10	kV/kV	0.54	0.42
Jimenez et al. (2019)	Breast	20	kV/kV	0.89 (0.38)	0.59 (0.25)
Gierga et al. (2008)	Breast	12	kV/kV	0.71 (0.19–2.4)	0.49 (0.12–1.8)

Overall, the difference in registration error between MixR and 3-point alignment was ≤ 0.2 cm for 11 of 18 cases, and ≤ 0.4 cm for 15 of 18 cases, although it did exceed 1.0 cm for one patient. For the proton cohort, the null hypothesis of the equivalence test was not rejected, meaning that the data cannot rule out a difference greater than 0.3 cm (the cutoff to obtain a significant result was 0.5 cm). For the photon cohort, the equivalence test was rejected, indicating that a difference of less than 0.3 cm was significant (the lower bound was not rejected indicating MixR could be better than SOC by more than 0.3 cm).

**Fig. 3:**
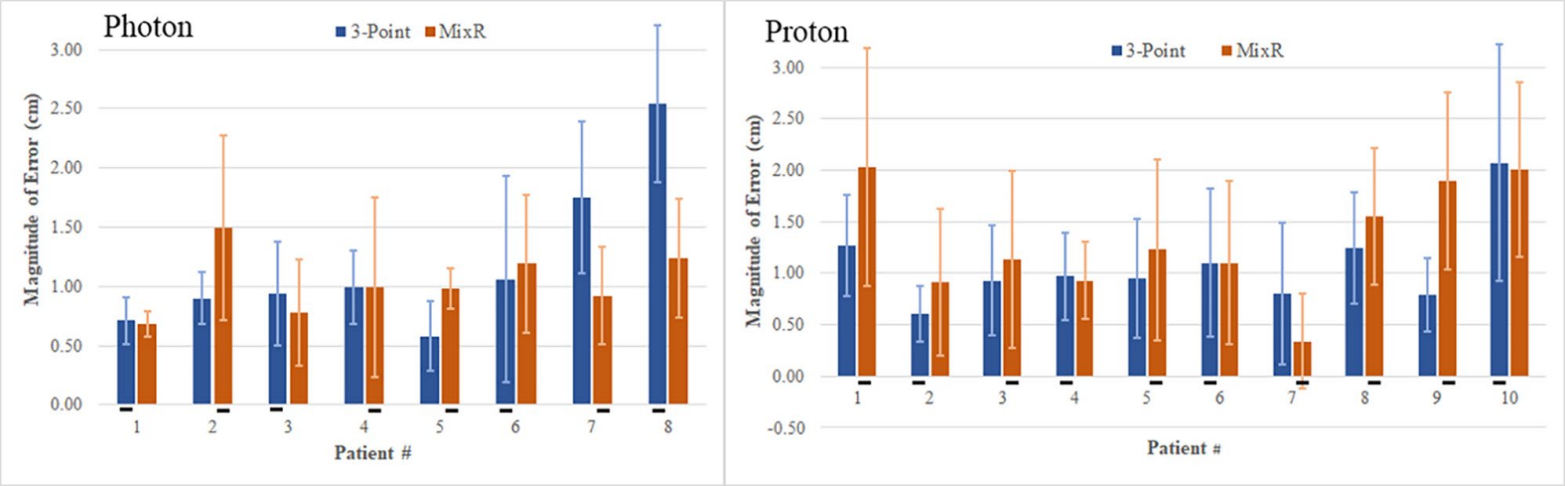
Magnitude of the average 3D error vector and standard deviation (cm) for each patient included in the study as categorized by modality and setup technique. Underlining on the x-axis represent the setup technique used for fractions 1–5. (mixed-reality; MixR)

The magnitude of the average 3D vector for each patient is shown in Fig. 3. As averaged across five fractions, the most accurate setup when using the MixR technique was 0.34 cm for the proton cohort and 0.68 cm for the photon cohort. Conversely, the least accurate setups had 3D vector magnitudes of 2.03 cm and 1.49 cm for the proton and photon cohorts, respectively. There was no linear relationship between 3D vector magnitude and patient weight, except within the photon cohort when using 3-point alignment where the R value was 0.77. Notably, patients 7 and 8 within the photon cohort were two of the largest patients in the study, and had, correspondingly, very large 3D vector magnitudes when using 3-point alignment. For these cases, MixR did much better, highlighting the potential benefit of the technique for correcting gross alignment errors. There were, however, a number of other cases where a MixR-based setup recorded a 3D vector magnitude in excess of 1.5 cm.

Nine different therapists participated during the clinical trial, with two of those individuals becoming de-facto super-users based on their repeated involvement in the study where they developed enhanced proficiency with the MixR system. These two therapists, one working with protons and the other with photons, participated in approximately 75% of the cases within their respective modalities. Aside from the absence of a super-user in the two longest cases within the proton cohort, there were no discernable patterns based on the involvement of one of these individuals. Throughout the study, no physiological issues such as headaches, nausea, or eye fatigue were reported by those who wore the HoloLens 2 device.

## Discussion

To our knowledge, this represents the first clinical use of MixR for patient setup in radiation oncology using a head-mounted display like the HoloLens 2. As noted previously, our initial work in this area, as well as a limited number of other studies, suggests registration errors of 0.3–0.6 cm for MixR-based setups using rigid, geometric or anthropomorphic phantoms [5, 8–10]. The additional error observed in this study is due, in part, to the challenge of moving from a rigid to a non-rigid setting. This is evident in that 3-point alignment, a technique with high fidelity in rigid scenarios, also struggled to produce accurate results during the trial. Due to the limited number of patients, it is difficult to explain the variation in the data and why certain patients appeared to benefit from MixR while others did not. Potential confounding factors were controlled as best possible including room lighting, color and texture of the patient hologram, experience level of the user, which HoloLens 2 device was used, the battery power of the device, and the steps involved in the workflow.

While not fully explanatory, a number of interesting observations were made during the study. First, when two users participated in a MixR-based patient alignment they often perceived the need for the same shift regardless of where each user was standing. This indicates a level of consistency between the HoloLens 2 devices, how they display patient holograms, and also the usefulness of marker-based tracking that allows two systems to synchronize their displays without the need to directly communicate with each other. As a limitation of the study, we did not consistently track when more than one user was involved in the holographic alignment process. This could have affected the registration time due to extended back and forth discussion between users. A broader exploration of MixR-based alignment via multiple users is needed and something we plan to look at in future studies.

Second, there were several occasions where a user visualized the need for a patient adjustment but had trouble positioning the patient in a way that accurately matched their hologram. This could be related to the position of the patient on the wing board, though effort was made to keep this relationship consistent as both the patient and wing board were visible as holograms. It could also indicate deformation in how the patient’s hologram was perceived, a condition observed in prior work and possibly related to the vergence-accommodation conflict, a known issue in optical-see-through systems that forces the brain to adapt to conflicting visual cues based on the use of a fixed focal plane within a truly, three-dimensional environment [11]. Third, there were occasions where the hologram appeared “off” to the user and this premonition coincided with a poor registration. For future work, it would be interesting to track the confidence level of each user during the registration process to see if this correlated with actual results. In this way, it might be easier to identify commonalities in poor registrations. Finally, both tracking mechanisms exhibited some offset as perceived when viewing the physical/holographic cube overlay. While expected for marker-less tracking in a dynamic environment, the performance of the optical, marker-based tracking showed room for improvement, something we are looking to address by transitioning to infrared (IR) -based tracking in the future.

In many ways, the MixR approach described in this study mimics the type of setup available with SGRT [12]. In both cases, the patient’s surface, as derived from their simulation CT scan, is used as a surrogate for the alignment of the target with the RT device. Of note, the MixR approach does not yet support intra-fraction motion monitoring as available with current SGRT systems. Several studies have quantified the benefits of SGRT in comparison to 3-point alignment for patients with tumors of the breast and chest wall (Table 3). The best analog to the current study is Stanley et al. in which CBCT was used to assess registration error in patients with breast cancer [13]. In that study, the average setup error when using 3-point alignment was 1.4 cm; this was subsequently reduced to 0.6 cm when using SGRT. Several other studies have further established the accuracy of SGRT in the breast at 0.2–0.6 cm when quantified using orthogonal kV or portal MV imaging [14–17]. Notably, there is limited information for cases involving the regional lymph nodes, with only one of the aforementioned studies including patients treated for such disease [14].

While similar in nature, MixR offers several advantages not presently available with SGRT. Notably, MixR affords direct observation of the patient and their reference surface without the need for ceiling mounted sensors or room monitors. As such, line-of-sight obstruction issues, limitations related to field-of-view (FOV), and poor ergonomics are all eliminated. Additionally, by designing the system for an off-the-shelf, portable device like the HoloLens 2, it may be possible to significantly reduce cost and improve access to this type of surface-guided setup.

To realize these benefits, MixR must eventually achieve the level of accuracy and efficiency demonstrated by modern SGRT systems. This study represents a first step along this path by demonstrating the feasibility of the concept of MixR for RT setup based on similarity with a traditional 3-point alignment in a limited setting. We recognize that the current technology is not yet ready for clinical implementation, and based on the results and the observations described herein, we have identified three areas where further advancement is needed. First, better tracking is required to both improve the initial registration of the hologram to the treatment device, and also to stabilize the position of the hologram during the dynamic aspects of patient setup. Much of the early work in MixR-based navigation/registration has relied upon optical trackers (e.g., QR codes, ArUco markers) as implemented via the Vuforia SDK or custom software [18, 19]. In controlled experiments, accuracy is reported at 0.1–0.2 cm [20], which is a difficult starting point for RT applications trying to achieve similar levels of accuracy during patient alignment. Notably, surgical navigations systems rely almost exclusively on IR-based tracking where reflective markers are attached to both the patient and surgical tools and tracked via external sensors. Recent studies have explored IR-based tracking using the time-of-flight sensors available on the HoloLens 2 and reported sub-millimeter accuracy [21, 22]. The authors of these studies highlight the improved resolution of IR-based tracking and its lack of sensitivity to ambient lighting conditions. Based on these findings, we are currently in the process of converting HoloMatch from optical to IR-based tracking using the open source code provided by Martin-Gomez et al. [22]; we are further developing individual IR-tracking markers for each of the treatment platforms used in the current study.

In addition to better tracking, quantifiable feedback is an important feature lacking in many MixR applications. It is both a benefit and limitation that an object is registered to a hologram by visually assessing the difference between the object’s surface and its reference, virtual surface. Direct, eye-level inspection is natural to a human observer but there is a limitation as to the level of detail that can be perceived. To quantify this difference, a virtual surface must first be extracted, in real-time from the physical object such that two virtual surfaces can be compared mathematically. The HoloLens 2 has the capability to extract a spatial mesh using its time-of-flight range sensors. We have attempted to utilize this mesh, along with computational raycasting to measure the difference between a physical and virtual surface and display it to the user in a color-coded fashion (see Supplementary Material). Unfortunately, the extracted mesh was too coarse and did not produce results reliable enough to warrant inclusion in this study. It is possible that we have not fully unlocked the potential of the HoloLens 2 for this purpose. Secondarily, there are several external systems (such as those employed in SGRT) that could provide a high-resolution spatial map of the patient to a wearable, MixR device in order to quantify the necessary shift and display it to the user numerically or by color-coding areas of the patient that need adjustment. This latter method would negate many of the advantages of the MixR system for improving portability and reducing cost but would maintain, in a full or limited fashion, those advantages related to FOV, line-of-sight obstruction, and ergonomics.

Finally, perceptual issues, like the aforementioned vergence-accommodation conflict, must be addressed either through hardware design or by better understanding how these issues manifest during patient alignment (see [23] for in-depth discussion of perceptual issues in MixR). The Magic Leap 2 device (Magic Leap, Inc., Plantation, FL), for example, tracks pupil dilation to better assess rendering needs and correct binocular image misalignment. Going in another direction, the video-pass-through system, Varjo XR-4 (Varjo Technologies Oy, Helsinki, Finland) utilizes 20 MP pass-through cameras to create MixR environments within a virtual reality (VR) setting. The system utilizes a gaze-driven autofocus to address accommodation issues in an attempt to mimic human vision. These are just a few examples of how the technology behind MixR is maturing and our expectation is that many of the issues encountered in this study are solvable and will eventually lead to improvements in overall accuracy.

## Conclusions

In this study, MixR visualization was implemented in a first-in-human clinical trial to assess the feasibility of the technique for patient setup in breast or chest wall RT. Registration accuracy, registration time, and IGRT utilization were compared with a traditional 3-point alignment in both photon and proton RT. Results in 18 patients showed minor differences between the two techniques indicating that MixR for patient setup is possible with current technology at the level of accuracy and efficiency provided by a 3-point alignment. Further developments in spatial tracking, quantifiable feedback, headset technology, and a better understanding of the perceptual challenges posed by MixR are needed to achieve a similar level of accuracy as provided by modern SGRT systems.

## Electronic supplementary material

Below is the link to the electronic supplementary material.

Supplementary Material 1

## Data Availability

No datasets were generated or analysed during the current study.

## Abbreviations

CBCT: Cone Beam Computed Tomography
FOV: Field of View
IGRT: Image-Guided Radiotherapy
IR: Infrared
MixR: Mixed-Reality
RT: Radiotherapy
SGRT: Surface-Guided Radiotherapy
SOC: Standard of Care
TOST: Two One Sided T-Test
VR: Virtual Reality
VSLAM: Visual Simultaneous Localization and Mapping
