# Supplementary material for: First-in-human trial using mixed-reality visualization for patient setup during breast or chest wall radiotherapy
Source: Radiat Oncol. 2024 Nov 18;19:163. doi: 10.1186/s13014-024-02552-0 (PMC11574990; doi:10.1186/s13014-024-02552-0)
Supplement: Supplementary file 1 — Supplementary Material 1 [file 13014_2024_2552_MOESM1_ESM.docx]

**Supplementary Material**

**
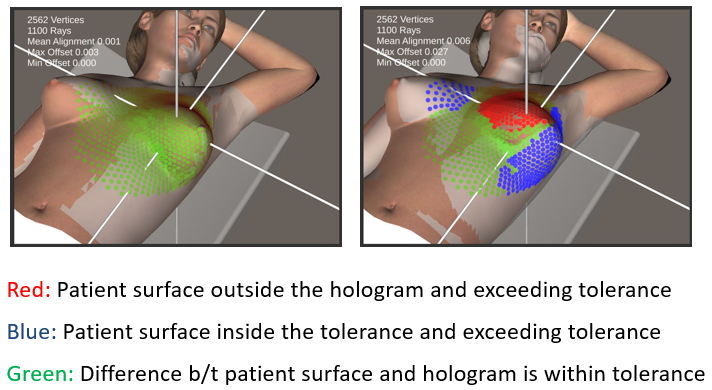
**

**Figure 1.s.** In this method, computational raycasting is used assess the overlap between the patient’s hologram (derived from their simulation CT scan) and the spatial mesh (derived from the patient’s physical surface at the time of treatment). Here, the spatial mesh is constructed as a virtual object via time-of-flight range sensors located on the HoloLens device. Evenly spaced rays are projected towards the target. The intersection points between a given ray and the patient’s hologram and a given ray and the spatial mesh are established. If the difference between these points is larger than a specified tolerance, the intersection point between the ray and the spatial mesh will display a specified color.
